# Cellular transfer and AFM imaging of cancer cells using Bioimprint

**DOI:** 10.1186/1477-3155-4-1

**Published:** 2006-01-22

**Authors:** JJ Muys, MM Alkaisi, DOS Melville, J Nagase, P Sykes, GM Parguez, JJ Evans

**Affiliations:** 1 Department of Electrical and Computer Engineering, University of Canterbury, Private Bag 4800, Christchurch, New Zealand; 2 MacDiarmid Institute for Advanced Materials and Nanotechnology, University of Canterbury, Private Bag 4800, Christchurch, New Zealand; 3 Christchurch School of Medicine and Health Sciences, University of Otago, Private Bag 4345, Christchurch, New Zealand

## Abstract

A technique for permanently capturing a replica impression of biological cells has been developed to facilitate analysis using nanometer resolution imaging tools, namely the atomic force microscope (AFM). The method, termed Bioimprint™, creates a permanent cell 'footprint' in a non-biohazardous Poly (dimethylsiloxane) (PDMS) polymer composite. The transfer of nanometer scale biological information is presented as an alternative imaging technique at a resolution beyond that of optical microscopy. By transferring cell topology into a rigid medium more suited for AFM imaging, many of the limitations associated with scanning of biological specimens can be overcome. Potential for this technique is demonstrated by analyzing Bioimprint™ replicas created from human endometrial cancer cells. The high resolution transfer of this process is further detailed by imaging membrane morphological structures consistent with exocytosis. The integration of soft lithography to replicate biological materials presents an enhanced method for the study of biological systems at the nanoscale.

## Introduction

Currently, optical microscopy techniques are the primary method for cell surface visualization, with microscopic characteristics of cells traditionally used for diagnosis and classification of cancers [1]. However, because the differences in characteristics can be subtle, accurate detection can be challenging and ambiguous [2]. A drawback of analysis using focused light microscopy is the fundamental diffraction limit, which at its optimum imposes an attainable spatial limit of 180 nm in the focal plane and 500 nm along the optical axis [3,4].

Imaging tools, such as the atomic force (AFM) and scanning electron (SEM) microscopes, are investigated for their ability to provide topographical information at a resolution far superior to optical methods [5,6]. Despite having the potential to image numerous diseases, cancers and pathogens, nanoscale analytical tools have not been efficiently utilized in mainstream biological research. Difficulties associated with imaging soft, living biological material in situ is challenging and remains a delicate and time consuming task, which while effective, is inefficient for the analysis and evaluation of large cell populations [7]. Predictions on the attainable resolution when imaging cell surfaces by AFM in liquid, regardless of living or fixed, is generally considered in be in the order of 50–500 nm [8-10]. Resolution limiting factors [11] include cell-to-substrate attachment, cell type, topographic complexity, surface composition and tip indentation into the soft biological material. In a probing-based imaging environment soft biomaterials are susceptible to structural movement and deformation caused by intermittent contact by the sharp AFM tip [12]. The tip apex is a crucial resolution limiting factor in AFM investigations and previous biological analysis has been limited to blunter probes for fear of membrane penetration [13].

Time consuming preparation procedures used for air and vacuum imaging environments require dehydration and fixation, which can also cause deformation and artifacts [14]. Although fluid-based AFM or SEM imaging attempts to address these issues by maintaining physiological conditions, factors such as scanning time, probe or electron interaction, and dampening effects are difficulties limiting these useful techniques. Consequently, nano-imaging as an analytical tool in biology remains under-utilized.

In the semiconductor industry, lithography enables the high resolution pattern transfer for the fabrication of nanoscale structures and devices. Recently, nanoimprint, a form of soft lithography, has been added as a candidate for next generation lithography; a successor to photolithography for pattern replication in the manufacturing of integrated circuits [15,16]. Soft lithography functions by contacting a structured template into a soft liquified polymer material, enabling a permanent replica to be fabricated after curing.

By using a technique, termed Bioimprint™, biological cells are directly integrated with soft lithography fabrication processes to create cell impressions in a robust storage medium for subsequent analysis using nano-imaging tools. In the process, a biocompatible liquid polymer is brought into contact with a cell before curing to create a negative replica.

This paper presents an alternative method for studying biological cells using a Bioimprint™ technique with AFM analysis, to enable the high-resolution replication and imaging of the surface topography of human endometrial cancer cells. As non-malignant endometrial cells were not immediately available as controls this work represents a preliminary study.

## Results

**Figure 1:**
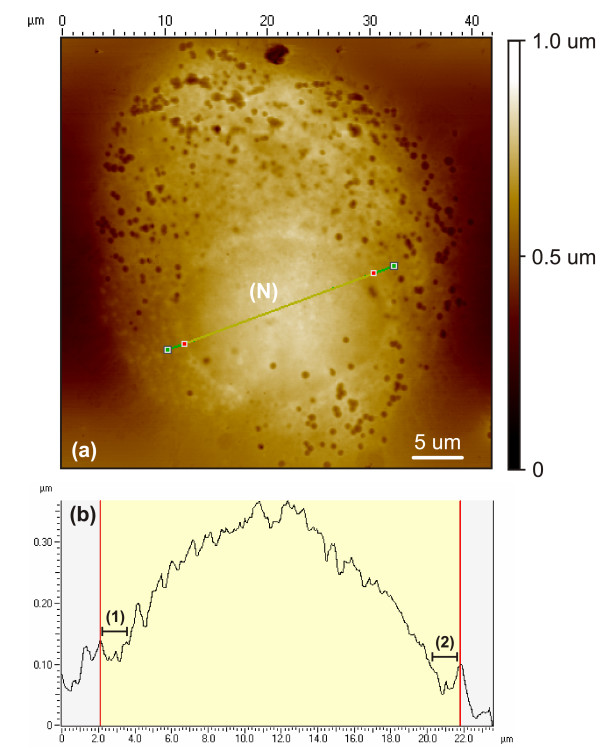
(a) AFM image of the positive replica of a 40 *μm *endometrial cancer cell created by digitally inverting its corresponding polymeric impression. The micrograph shows numerous dimple depressions scattered and concentrated around a nucleus (N) form, which is visible by the conformation of the membrane around it. (b) A scope trace focused on the membrane above the nucleus details the indentation (1),(2) profiles at locations on the membrane surrounding the nucleus, indicating cell dehydration.

The ability of the Bioimprint™ process to accurately replicate and transfer cellular topography into a Poly (dimethylsiloxane) (PDMS) polymer is investigated. Figure 1(a) shows a Bioimprint™ replica of a malignant endometrial cell, which is positively inverted to achieve a digital transpose of the negative replica or 'impression' made by the cell during imprinting. The replica presents visible cellular features on both micron and nanoscales. Throughout the image, numerous dimple depressions, which have a mean width and depth of 820 nm and 360 nm, respectively, are seen located on the membrane. Though these features appear to be too large to be fusion pores, they are potentially associated with exocytosis.

Exocytosis has been described in numerous ways with wide speculation governing both the underlying mechanisms driving membrane fusion as well as membrane topology. It is accepted that fusion begins by a granule or vesicle from within the cell docking at the membrane to release its contents. The manner by which the contents of the granule are released remains debated, and there are arguments supporting both total and transient fusion as described by 'fuse-and-collapse' and 'kiss-and-run' mechanisms, respectively. Visual verification has however been limited, partly due to the difficulties with imaging living or structurally intact cells at high resolution, and the lack of well-defined protocols and methods integrating nano-imaging tools with biology. The dimple model [17-21] predicts that exocytosis is initiated by membrane fusion; in which, a scaffold built into the membrane dilates to create a dimple site, where subsequently an underlying granule docks to create a fusion pore and release its contents. While other models [22,23] for membrane fusion exist this remains one of the most convincing and well documented models for exocytosis.

An additional feature depicted in Fig. 1(a) is the outline of a spherical form impacting on the cell membrane, which is assumed to be the nucleus (N). In Fig. 1(b) a scope trace reveals the impact of the underlying nucleus on the membrane, causing a distorted effect indicated by points (1),(2). Weyn et al. [24] have investigated the dehydration effects on malignant mesothelioma cells by AFM and reported a much harder and uniform indentation profile over the entire cell, whereas hydrated cells have a more rounded and smooth surface. Nuclei collapse was also a possible feature resulting from dehydration effects and though some cells demonstrated nuclei submersion, this did not occur in every cell imprinted.

**Figure 2:**
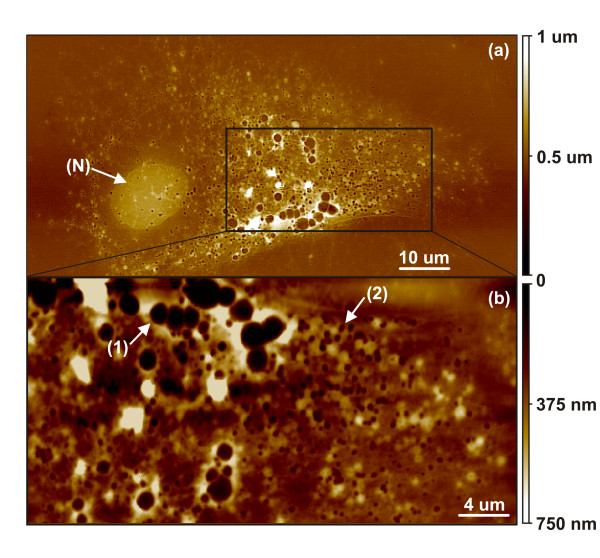
(a) Large-area (110 *μm *wide) AFM scan of a positive replica made from an endometrial cancer Bioimprint™ impression, illustrates a rounded nucleus (N) beneath a membrane containing numerous dimple depressions of varying sizes. (b) A 40 *μm *wide magnification of the membrane reveals two types of depressions; deep and wide (1) as well as more abundant smaller and shallower (2) pits.

The impact of the location of the nucleus on the formation of dimple depression sites on the membrane is further evident in Fig. 1(a), where they are seen predominantly concentrated at areas around the nucleus. This is reinforced in the AFM positive replica of the endometrial cancer cell shown in Fig. 2(a), where the majority of dimple depressions are scattered around the nucleus (N). Here, in contrast to Fig. 1(a), the nucleus appears well hydrated and as a uniform rounded structure with no indentation profile or submersed effect. In Fig. 2(b), a 10 *μm *image selectively focused on an area of the membrane is seen saturated by both spherical larger and more numerous smaller depressions, as shown by points (1) and (2), respectively. This illustrates the significant variation in the size of depression sites seen at the membrane.

**Figure 3:**
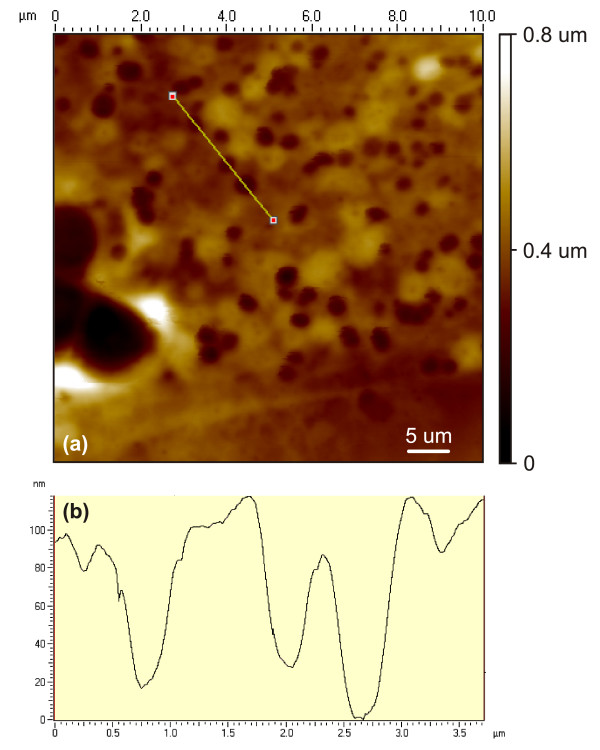
(a) A 10 *μm *AFM height image taken from a Bioimprint™ positive replica showing numerous pits scattered on the membrane of a malignant endometrial cell. (b) A scope trace taken across the membrane illustrates three smaller dimple depressions having opening widths of approximately 600 nm and 100 nm deep and formed as concave submersions.

An additional benefit of the AFM is its ability to accurately sense 3-D topography with a high degree of contrast. In radiation scattered devices, such as light microscopy, the contrast is weak and the Z-dimension is often disregarded as analytical and quantitative evidence in diagnosis, or when evaluating cellular function. This is illustrated by the scope trace in Fig. 3(b) measuring the cross-section of 3 smaller dimples in (a). The dimple depressions are seen having an average diameter of 600 nm and depth of 100 nm, whereas, the larger pits appear much deeper.

**Figure 4:**
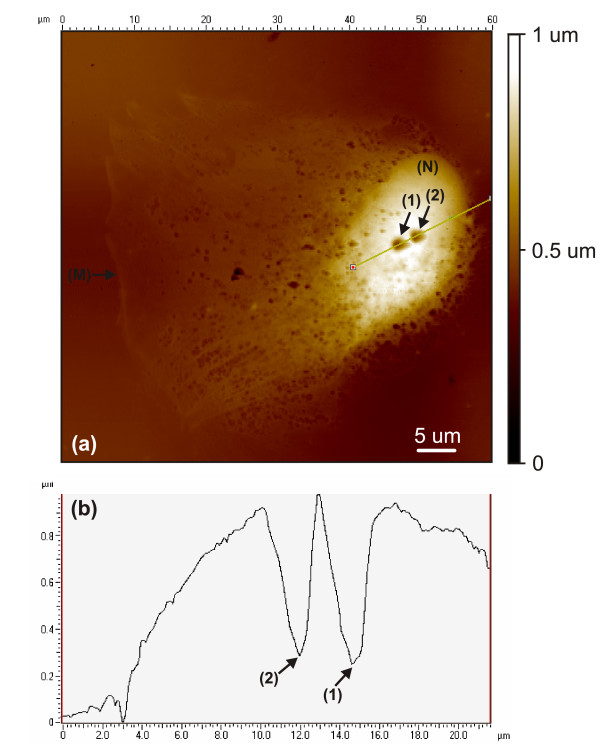
(a) A Bioimprint™ positive replica of the impression made from a 50 *μm *endometrial cancer cell, shows the membrane extending leftwards from a 1 *μm *tall rounded nucleus (N) body, which is seen to contain two large ruptured depressions (1),(2). (b) A scope trace examining these ruptures (1),(2), shows them to be approximately 3 *μm *wide and submersing deep within the cell.

Observations in this study suggest that the cell replicas imaged had diverse morphologies potentially caused by cancer mutations, which act to deform and distort the cell structure in several ways [25,26], and often result in complex and varying cellular forms. Even considering the potential artifacts caused in the cell replication process there is large variability in the shape and locations of distinguishable cell features. Figure 4(a) reinforces this by showing a positive Bioimprint™ replica of an endometrial cancer cell bearing a different appearance and nucleus arrangement from those presented previously. A rounded nucleus (N) is seen clearly offset to the right of the cell, with the membrane extending leftwards. Again, numerous depressions are seen located on the membrane around the nucleus, but especially apparent are two large pits located on the membrane directly above the nucleus. The scope trace in Fig. 4(b) shows the depressions formed as ruptures, approximately 3 *μm *wide and extending at least 700 nm deep within the cell. The shape and actual depth of the rupture is difficult to accurately measure due to limitations associated with the imaging tip, which has resulted in an image that reflects the profile of the imaging tip rather than of the rupture.

**Figure 5:**
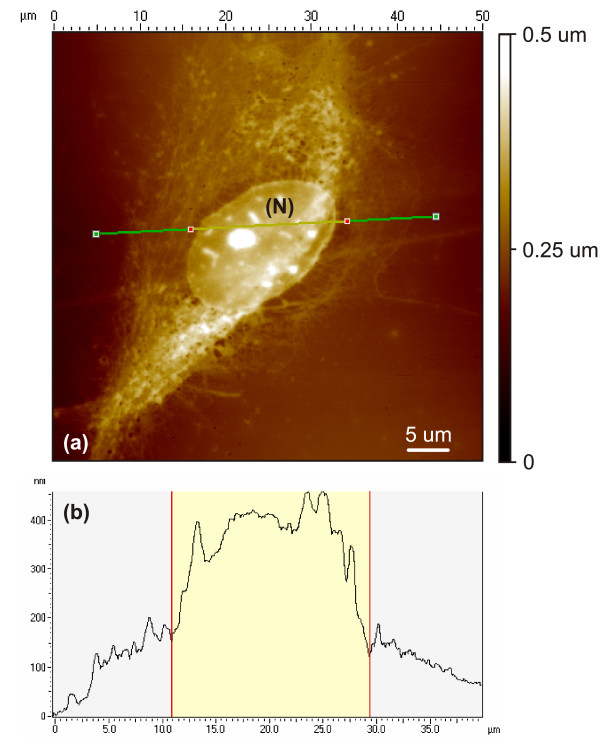
(a) A 50 *μm *AFM scan of a Bioimprint™ positive replica showing a malignant endometrial cell with a nucleus (N), seen distinctly protruding from above the membrane level. (b) In a scope trace the nucleus is seen to be 18 *μm *wide, extending approximately 300 nm above the membrane.

Further illustrating the range and variation of cell morphologies, and the potential effect the thick layer of polymer has in generating nuclei artifacts is illustrated in Fig. 5(a): An AFM image of a Bioimprint™ positive replica shows a 40 *μm *malignant endometrial cell with a unique nucleus (N) form, which appears to be distinctly separated from the membrane. A scope trace in Fig. 5(b) quantitatively illustrates the 18 *μm *wide nucleus, which is seen extending sharply by ~300 nm above the surrounding membrane level. While other cell types imaged do not display such variation in nuclei form and membrane structure, without non-malignant controls it remains uncertain whether these are an artifacts induced from the Bioimprint™ process or cellular properties that are characteristics of cancer.

## Discussion

Much is yet to be known about the nature of endometrial cancer cells and until now there has not been a reliable, simple method for visualizing cell topography in air, at high resolution without fixation and dehydration. Being able to directly view membrane structures regulated by exocytosis will enable researchers to analyze the secretory nature and response of cells, yielding insights into drug responses and effects. Considerable variability in the sizes of dimple depressions and ruptures, as well as dynamic formation and grouping of these structures around the nucleus, illustrates that cells have diverse morphologies.

There was an inconsistency in the degree of deformities in the cancer cells: Reasons for the varying nuclei forms seen could potentially be explained by the weighted force of the polymer, acting to press the membrane down and casting the nucleus as a structure protruding from the cell. However, the ability to view and potentially characterize the effects of cancer mutations at both micron and nanoscales presents a remarked improvement over conventional optics. In addition to the high resolution imaging enabled by the AFM, the ability to image accurately in 3-dimensions presents a significant advantage over radiation scattered imaging devices. The sensitivity of the AFM and precise transfer of cell topography into a polymer provides a method with the ability to overcome the current difficulties of imaging biological materials by the AFM.

Advantages of Bioimprint™ extend beyond simple lithographic process replication, with benefits such as prolonged storage and analytical adaptability without lose of resolution. Additionally, being a non-biohazardous substance, impressions of pathogenic material, infected cells and other biological samples can be transported or exchanged for analysis without contamination concerns. This would reduce the need for complicated and lengthy documental approval, which is required by governments and institutions, and would facilitate out-of-house analysis. Further gain is the ability to keep patient records using an indirect specimen, without the need for expensive storage or contamination equipment.

Usefulness of Bioimprint™ can especially be realized when used as a complementary technique with conventional optical imaging, and as alternative method for those requiring strict sample preparation, such as chemical fixation and dehydration in order to visualize cell topology by AFM [27] or SEM [28]. Such applications could employ immunohistochemistry methodology on the actual cell, prior to replicating the cell topography using Bioimprint™. This combined physical and chemical approach may yield a better understanding into cell functionality and mechanics.

Artifacts in the form of bubbles caught trapped between the cell and polymer and ripping of the cell membrane were also observed and readily identifiable. A crucial factor in imprinting the cell structure is the amount of fluid remaining on the surface prior to polymeric application. Absence of a thin layer of medium above the cells inevitably causes aridity, and resulted in many Bioimprint™ replicas displaying characteristic nucleus dehydration artifacts. On the other hand, too much fluid will create an interfacial layer that impedes the transfer of high resolution features.

A limitation in the current methodology used to fabricate imprints is that the process lacks the control required for fabricating accurate and consistent cell replicas. Imprinting conditions are too slow and a large proportion of the cell population failed to be accurately replicated due to significant dehydration effects. Using the heat curing PDMS polymer, the replicas are in fact a 'time-averaged' rather than a 'single-shot' capture impression, in which the polymer conforms to the cell structure. Artifacts are inevitably being introduced by the cellular response to the polymer and curing conditions, which are most noticeably shown by the affects on the nucleus.

Currently, efforts are being concentrated around developing a rapidly U.V. curable polymer formed as a thin pre-spun layer, which is imprinted rather than poured above the cells in a bulk [29]. While the U.V. light is undoubtedly detrimental to cell physiology, the time taken to replicate is 100-fold shorter than the conventional PDMS composite, enabling a more accurate representation of the living cell to be replicated. Preliminary results show a reduction in the number of cells displaying nuclei submersion or dehydrating effects and an improved resolution transfer.

## Conclusion

A soft lithographic technique for creating replica cell impressions with nanoscale information transfer has been introduced and tested on human endometrial cancer cells. By creating a cell 'footprint' in a stable solidified polymer, a permanent non-biohazardous record can be kept and analyzed at high resolution using the AFM. Bioimprint™ overcomes many of the inherent difficulties associated with cellular imaging by AFM and advances their integration as investigative tools in biology. With visual verification ultimately being the mainstay for cancer diagnosis, a method facilitating the use of imaging at potentially atomic resolution could be used more to characterize morphological abnormalities at the nanoscale. Though at this time, it is difficult to deduce if the varying shapes and forms are potential characteristics linked to malignant cell mutations, or if they are artifacts induced from the Bioimprint™ replication process. This study reports preliminary work in the areas of cellular replication and endometrial cancer cell imaging by atomic force microscopy.

## Methods

Human endometrial cancer cells were cultured in accordance with institutional guidelines of the Christchurch School of Medicine and Health Sciences, University of Otago, New Zealand, after ethical approval and appropriate informed consent. The preparation of cells were as follows: Endometrial adenocarcinoma tissues were harvested from women undergoing hysterectomy, and non-myometrial biopsies were taken from the opened uterus tumor area. Tissues were then digested in collagenase-A (1 mg/ml), and the cells dispersed, and cultured overnight in medium consisting of alpha-MEM containing 1 % penicillin/streptomycin, 0.1 % BSA and 10 % fetal calf serum.

**Figure 6:**
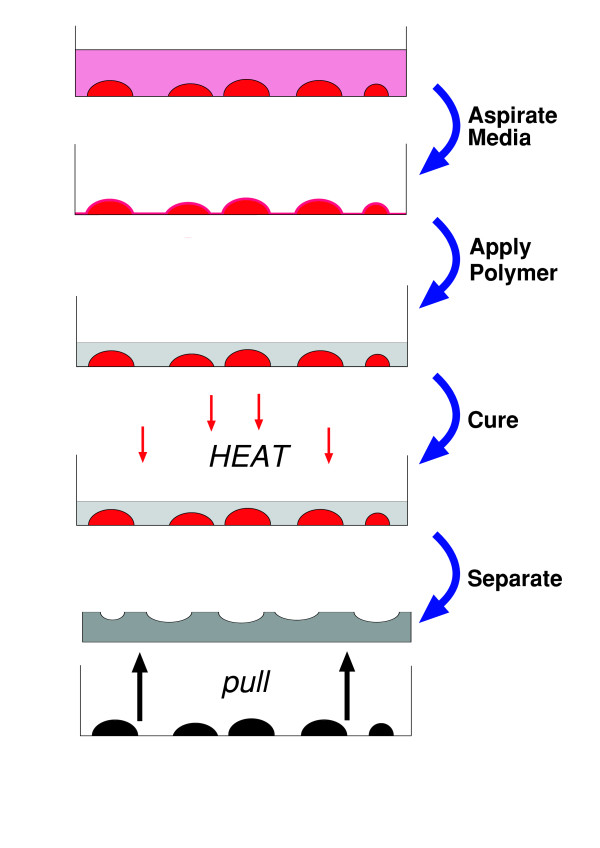
Bioimprint™ pattern transfer scheme for fabrication of negative cell replicas: Initially, all suspending medium is removed from cells attached to a Petri-dish, then a PDMS polymeric composite is poured over the cells and incubated. After curing, the hardened polymer is separated from the cells, washed and then the cell impression or mold is scanned by an AFM and digitally inverted to yield a positive replica matching the original cell orientation.

Prior to polymer application all incubation media was aspirated and samples were washed in physiological phosphate-buffered saline (PBS). The pattern transfer scheme for impression fabrication is illustrated in Fig. 6: Initially, Poly(dimethylsiloxane) (PDMS) (Dow Corning, USA) solution was mixed at a ratio of 10:3 of polymer to curing agent, the air was removed from the solution in vacuum and pre-cured for 2 mins at 95°C. Approximately 5–8 grams of composite was applied above the cells attached on a 5 cm plastic Petri-dish and immediately incubated in a 37°C oven for 2 hours. The thickness of the resulting polymer above the cells was typically between 2.5 and 5 mm. The attachment of cells to the substrate prevent features from being submersed completely within the polymer material, enabling an impression of the exposed surface of the cells to be made in the polymer. The mask was peeled off, washed in DIW ultra-sonic bath to remove any biological material attached and a final polymerization stage was completed in a 95°C oven for 2 hours.

The hardened Bioimprint™ impressions were analyzed by an AFM (DI 3100, Veeco Instruments, Santa Barbara, CA) in tapping mode using triangular non-contact cantilevers (NSC11, MikroMasch, Estonia), which were typically operated between 0.6–1 Hz at a resonant frequency of ~315 kHz with a nominal sub-10 nm radius of curvature and a force constant 48 N/m. To recover the original cell orientation, positive replicas are made by digitally inverting the AFM scans of the impressions/molds/negative replica, which were made by the cells when imprinted in the polymer.
